# UHR as a systemic sensor of cumulative heat exposure and subclinical cardiovascular injury: evidence from 64,088 adults

**DOI:** 10.3389/fpubh.2026.1825331

**Published:** 2026-05-07

**Authors:** Tao Cheng, Rui-Chen Cui, Jiu-Lin Guo, Zhen-Kun Zhao, Ping Zhang, Guo Tang, Tian-Shan Zhang, Lu Gan, Rong Yao

**Affiliations:** 1 Department of Emergency Medicine, Institute of Disaster Medicine and Institute of Emergency Medicine, West China Hospital, Sichuan University, Chengdu, Sichuan, China; 2 Department of Thoracic Surgery and Institute of Thoracic Oncology, West China Hospital, Sichuan University, Chengdu, Sichuan, China; 3 Information Center, West China Hospital, Sichuan University, Chengdu, Sichuan, China

**Keywords:** cumulative heat exposure, double-hit hypothesis, remnant cholesterol, subclinical cardiovascular injury, uric acid-to-HDL cholesterol ratio (UHR)

## Abstract

**Background:**

The increasing frequency and intensity of heatwaves threaten global cardiovascular (CV) health. While the link between acute heatwaves and clinical CV events is known, the effect of cumulative heat exposure on early, subclinical CV injury is poorly understood. We investigated the dose–response relation between sustained thermal burden and subclinical CV damage and identified the main metabolic-inflammatory pathways involved.

**Methods:**

In this large-scale, population-based retrospective cross-sectional study of 64,088 adults from Sichuan, China, we quantified cumulative heat exposure using new Cumulative Excess Heat Wave Index (CEHWI) based on apparent temperature (AT). Subclinical CV injury was assessed using a progressive cascade of biomarkers, including lipid remodeling markers (RC), Atherogenic Index of Plasma (AIP) and myocardial injury enzymes (AST), Lactate Dehydrogenase (LDH). We used multivariable generalized linear models and causal mediation analysis to evaluate 10 candidate metabolic-inflammatory mediators in metabolic, lipid and inflammation domains.

**Result:**

Higher CEHWI is associated with a dose-dependent increase in subclinical CV injury markers. Each unit increase of CEHWI was associated with elevated RC (*β* = 0.088 mmol/L), AIP (*β* = 0.019) and LDH (*β* = 2.069 U/L; all *p* < 0.01). Mediation analysis showed Uric Acid-to-HDL Cholesterol Ratio (UHR) to be the most important mediator explaining 73.7% of the total effect of cumulative heat on AIP (*p* < 0.01) and revealing a previously unknown metabolic–inflammatory axis. Subgroup analyses supported the Double-Hit hypothesis, indicating heightened vulnerability among older adults and hypertensive individuals. Hypertension acted as a “priming factor” that reduced the threshold of heat-induced metabolic collapse even for active clinical treatment.

**Conclusion:**

Short-term cumulative heat exposure(7-day CEHWI) is strongly associated with subclinical CV injury, with the association largely mediated through a UHR-related metabolic-inflammatory nexus. These findings underscore the necessity of transitioning toward cumulative intensity monitoring in climate-health monitoring. Integrating UHR as a “biological thermometer” in early warning systems and precision prevention strategies for metabolically vulnerable populations, such as metabolic monitoring during extreme heat periods, are public health imperatives in warming world.

## Graphical abstract

**Figure:**
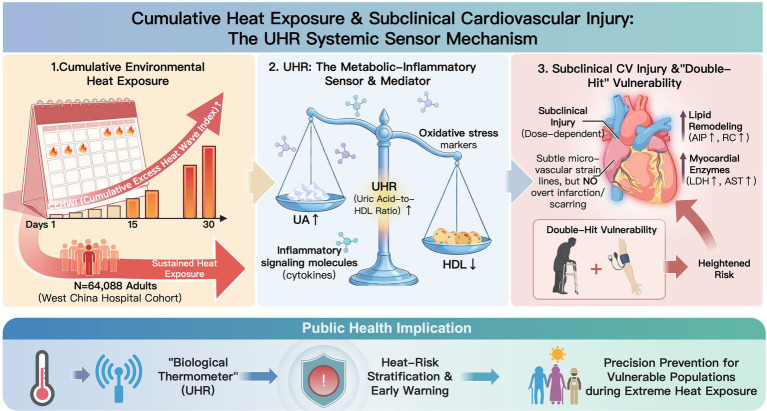

## Highlights

- Subclinical CV injury displays a dose-dependent escalation with cumulative heat.
- The multi-dimensional CEHWI provides a robust proxy for sustained thermal burden.
- UHR emerged as the predominant mediator, explaining 73.7% of the total effect on AIP.
- Hypertension acts as a “priming factor” for heat-induced metabolic collapse.
- UHR serves as a low-cost “biological thermometer” for heat-risk stratification

## 1 Introduction

As global warming continues, extreme heat events have moved from occasional anomalies to systemic public health threats (1–4). The Lancet countdown reports an 85% rise in heat-attributable CV mortality over the last 20 years (5–7). Although short term heatwaves may have link to acute clinical end points such as myocardial infarction or stroke, these endpoints are late stage manifestations of a much larger range of environmental harms (8–12). One of the key but not so explored research areas is the subclinical stage of cardiovascular disease, where cumulative heat exposure leads to gradual physiological dysregulation before obvious disease (13–15).

The current research on heat-related cardiovascular risk is dominated by four main limitations: (1) the focus is too much on clinical endpoints and not on subclinical damage (8, 13). Most studies focus on hard outcomes, thus overlooking early, potentially reversible changes in lipid metabolism and cell integrity before clinical events, which is important for early risk stratification. (2) The characterization of exposure is inadequate (10, 16). The simplistic metrics of exposure such as daily maximum temperature (the daily maximum temperature) fail to adequately represent the cumulative biological “dose” of heat. Indicators of frequency, intensity and duration (the CEHWI (17), for example) are theoretically more robust, but do not have validation in large real-world cohorts. Third, the theoretical pathophysiological framework is incomplete. Though systemic inflammation and oxidative stress are widely viewed as potential drivers, the metabolic pathways that translate thermal stress into measurable CV injury are not well defined (18–20). New evidence suggests that heat-induced dehydration and metabolic stress may disrupt the balance between uric acid production and lipid remodeling (21–23). This finding makes compelling arguments for investigating UHR (integrated biomarker of metabolic-inflammatory status) as a potential link (24–26). However, it remains to be empirically tested whether UHR mediates the “environment-cardiovascular” axis under cumulative heat stress. Fourth, there is a notable lack of research focusing on vulnerable subpopulations, particularly those with metabolic precursors such as obesity or dyslipidemia (8, 27). This gap hinders the development of targeted, precision-based protective strategies.

To address these gaps, we analyzed a Chinese health-screening cohort of 64,088 participants to examine the early cardiovascular effects of cumulative heat exposure. We used the CEHWI together with subclinical markers — RC, AIP, AST and LDH — to characterize patterns of subclinical injury (28–31). Furthermore, we systematically evaluated a panel of 10 candidate mediators, including systemic inflammation indices (for example, SIRI, SII and leukocyte-based ratios) and metabolic–lipid ratios (for example, TyG, PHR and UHR). By comparing their relative mediating strengths within the environment–cardiovascular axis, we aimed to identify the principal biological pathways by which thermal stress disrupts cardiovascular homeostasis. The study’s findings intend to provide a rigorous scientific basis for environmental risk management and personalized interventions amid rising global thermal instability.

## 2 Patient population and methods

### 2.1 Patient population

This retrospective cross-sectional study used the health-examination database of the Health Management Centre, West China Hospital, Sichuan University, and covered the period 1 February 2023 to 1 November 2024. Eligible participants were aged 18 years or older, had completed at least one comprehensive blood biochemistry panel, and had meteorological data available for the 7 days preceding their examination. Exclusion criteria were (1) age less than 18 years; (2) missing key exposure (meteorological) data; (3) incomplete cardiometabolic profiles required to calculate residual cholesterol or plasma atherogenic index. After screening, 64,088 participants remained for analysis. The study protocol was approved by the Institutional Review Board and adhered to the ethical principles of the Declaration of Helsinki (32).

### 2.2 Heat exposure assessment

Cumulative heat exposure for the 7 days preceding the health examination was assessed by combining the activity of each participant with local weather data. Daily environmental parameters (average, maximum, minimum temperature, relative humidity, air pollution) were calculated from the monitoring stations closest to each participant’s home or workplace (in the baseline registration records at the Health Management Centre). To account for human thermal stress, AT was calculated from ambient temperature and humidity (33, 34). AT was calculated using the Steadman formula:


AT=Tα+0.33×P−0.70×v−4.00

where Tα is air temperature (°C), P is vapor pressure (hPa), and *ν* is wind speed (m/s) (33).

The 90th percentile (P90) of AT during the study period was used as the threshold heatwave days. The exposure variables were calculated for the 7 days before each participant’s examination (in order to calculate the cumulative heatwaves). CEHWI was computed by summing daily AT values exceeding P90 over the 7 days, representing the cumulative heat exposure.

### 2.3 Cardiac outcomes, mediator, and covariates

#### 2.3.1 Outcome and mediator measurement

Cardiac outcome measures, representing subclinical cardiovascular injury, included RC, AIP, LDH, AST and cardio-metabolic index (CMI); these biomarkers reflect early metabolic and cellular changes associated with increased cardiovascular risk, including lipid remodeling (RC, AIP) and cellular stress/injury (LDH, AST). RC was calculated as total cholesterol minus LDL-C minus HDL-C (35). AIP was defined as log_10_(TG/HDL-C) (36). CMI was derived from standard formulae combining waist circumference and lipid ratios (37). AST and LDH were measured by standard automated enzymatic methods. Potential metabolic and inflammatory mediators comprised the UHR, the systemic immune–inflammation index (SII) and the systemic inflammation response index (SIRI). UHR was defined as serum uric acid (μmol/L) divided by HDL-C (mmol/L) (38). SII was calculated as (platelet count × neutrophil count) / lymphocyte count (39), and SIRI as (monocyte count × neutrophil count) / lymphocyte count (40).

#### 2.3.2 Covariates selection

Multivariable models were adjusted for a comprehensive set of covariates to minimize potential confounding: (1) demographic characteristics (age and sex); (2) lifestyle factors (history of smoking and alcohol consumption); (3) chronic conditions (documented diabetes, hypertension and sleep disorders); (4) psychological status (anxiety and depression); (5) anthropometric measures (weight, height, BMI, waist-to-hip ratio [WHR] and waist-to-weight index [WWI]); and (6) bone mineral density (BMD), classified as normal, osteopenia or osteoporosis by dual-energy X-ray absorptiometry (DXA). Information on specific antihypertensive medication types or dosages was not available in the health examination database.

### 2.4 Statistical analysis

Multivariable generalized linear regression models were used to examine associations between CEHWI and subclinical CV injury markers, adjusting for the covariates described above. Notably, to isolate the independent effect of thermal exposure, we rigorously adjusted for ambient air pollutants (PM_2.5_, PM_10_, O_3_, NO_2_ and SO_2_) and meteorological variables (for example, wind speed and vapor pressure) in sensitivity analyses. Results are presented as regression coefficients (*β*) or relative risks (RRs) with 95% confidence intervals (CIs). We conducted stratified analyses by sex, age, BMI, WHR, BMD, and co-morbidities (diabetes, hypertension, sleep disorders, anxiety and depression) to assess potential effect modification; interaction terms were tested by adding product terms to the models. Mediation analysis was performed using the R ‘mediation’ package (41) to quantify the potential mediating roles of metabolic (UHR) and inflammatory (SII, SIRI) biomarkers in the heat–cardiovascular association, using 5,000 bootstrap iterations to ensure robust interval estimation for the UHR-mediated axis. All analyses were performed in R (version 4.4.1; R Foundation for Statistical Computing, Vienna, Austria). Two-sided *p*-values < 0.05 were considered statistically significant. To assess the robustness of the prespecified 7-day CEHWI definition, we performed sensitivity analyses using alternative cumulative exposure windows (1, 3, 5, and 7 days before examination). For each window, CEHWI-based associations with key subclinical cardiovascular outcomes were re-estimated using the same modeling framework and covariate adjustment as in the primary analysis.

## 3 Results

### 3.1 Participant selection and baseline characteristics

**Figure 1:**
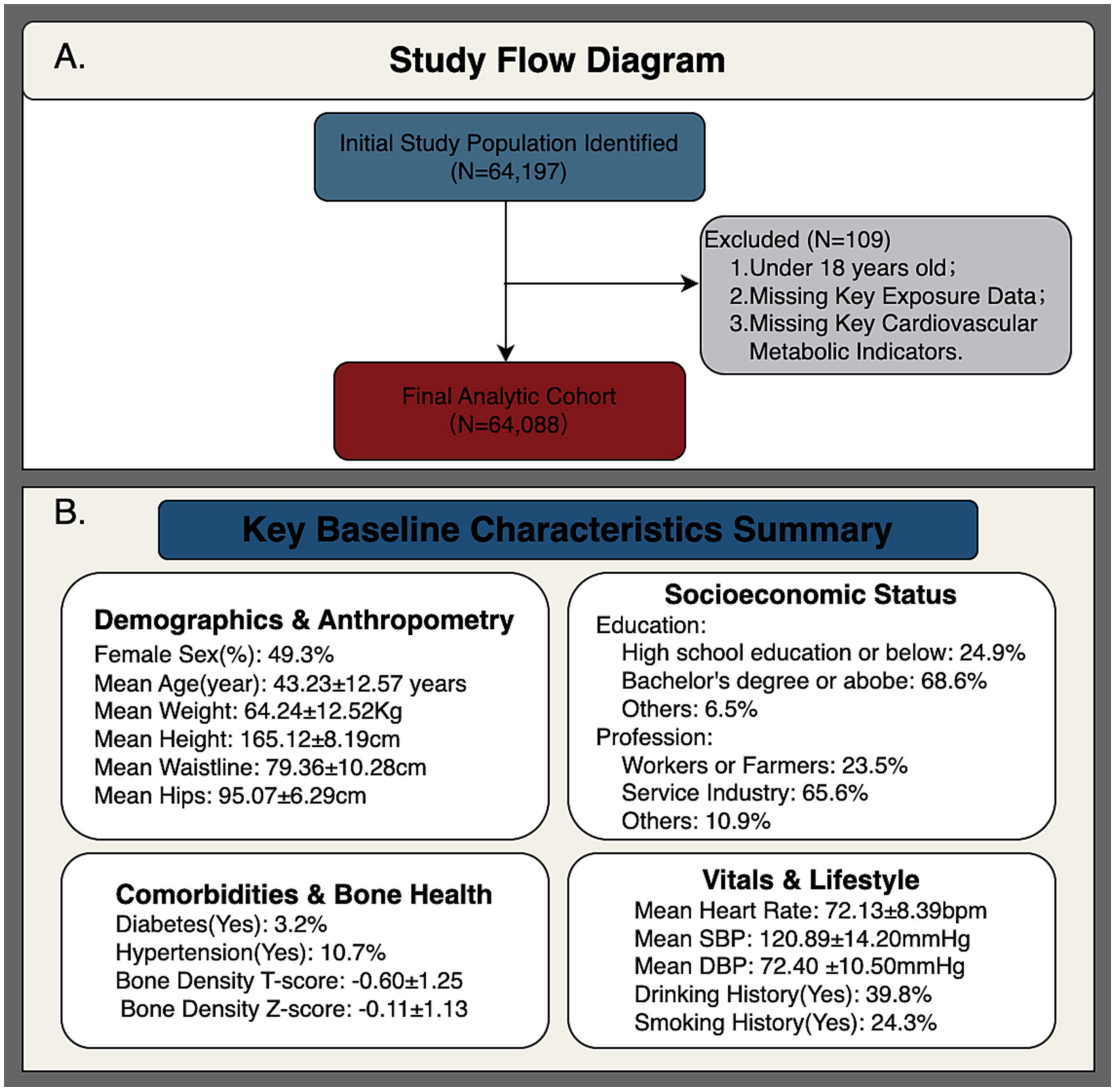
Participant selection and population profile. **(A)** Flowchart of the sequential inclusion/exclusion process for 64,088 participants. **(B)** Baseline demographic, clinical, and lifestyle characteristics of the study cohort.

Figure 1A shows the participant selection process, resulting in a final analytical sample of 64,088 individuals after excluding those with incomplete profiles or missing meteorological data. As summarized in Figure 1B, the cohort was predominantly middle-aged (mean age 43.23 years, SD = 12.57) with an approximately equal sex distribution (50.7% male). Educational attainment was high (68.6% university-educated) and most participants worked in the service sector (65.6%). Clinical measurements were within normal limits: blood pressure averaged 120.89/72.40 mmHg and heart rate 72.13 bpm. Anthropometry and bone mineral density (mean T-score −0.60) indicated a generally healthy baseline. The prevalence of chronic disease was relatively low, with hypertension at 10.7% and diabetes at 3.2%. Histories of alcohol use (39.8%) and smoking (24.3%) were recorded. In sum, this well-educated, urban, middle-aged cohort represents a robust sample for examining the subtle metabolic and CV effects of cumulative thermal strain.

### 3.2 Associations between heat exposure and cardiac outcomes

**Figure 2:**
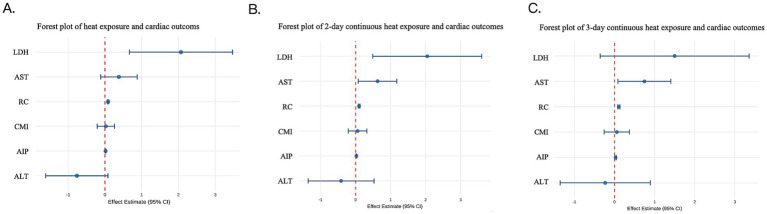
Summary effect estimates of cumulative heat exposure on subclinical cardiovascular injury markers across different durations. The forest plots show associations between the Cumulative Excess Heat Wave Index (CEHWI) and a panel of biomarkers—lipid-remodeling markers (RC, AIP), myocardial and hepatic enzymes (AST, LDH, ALT), and the cardio-metabolic index (CMI)—for three exposure windows: **(A)** 1-day, **(B)** 2-day and **(C)** 3-day continuous heatwave durations. The horizontal axis (x-axis) shows the adjusted effect estimates (*β*) with their 95% CIs.

Multivariable generalized linear regression showed significant associations between CEHWI and elevated subclinical CV injury markers (Table 1). In the single-day model, heat was significantly associated with higher RC (*β* = 0.088), AIP (β = 0.019) and LDH (*β* = 2.069; all *p* < 0.01), whereas the positive association with AST was not statistically significant (*p* = 0.133). The magnitude of these associations followed a clear duration-dependent pattern. Two- and three-day continuous heat exposure produced progressively stronger associations for RC (*β*max = 0.108, *p* < 0.01) and AIP (*β*max = 0.027, *p* < 0.01). AST became significantly raised after at least 2 days of continuous exposure (*p* < 0.05), while the association with LDH weakened by day 3 (*p* = 0.111). No significant associations were found for CMI in any time window (*p* > 0.05). Overall, these results indicate that cumulative thermal intensity, as measured by CEHWI, has a greater biological effect than transient peaks (Figure 2), with particularly pronounced effects on remnant cholesterol.

**Table 1:** Generalized linear regression of heat exposure and cardiac outcomes.

**Term**	**RC**				**AIP**				**AST**				**LDH**				**CMI**			
	**Est.**	**S.E.**	**Stat.**	***P***	**Est.**	**S.E.**	**Stat.**	***P***	**Est.**	**S.E.**	**Stat.**	***P***	**Est.**	**S.E.**	**Stat.**	***P***	**Est.**	**S.E.**	**Stat.**	***P***
Heat exposure (1 days)	0.09	0.01	8.37	<0.01	0.02	0.01	3.06	<0.01	0.38	0.25	1.50	0.13	2.07	0.72	2.89	<0.01	0.02	0.12	0.20	0.84
Continuous heat exposure (2 days)	0.10	0.01	8.55	<0.01	0.02	0.01	3.41	<0.01	0.63	0.28	2.23	<0.05	2.05	0.79	2.58	<0.01	0.06	0.13	0.42	0.68
Continuous heat exposure (3 days)	0.11	0.01	7.71	<0.01	0.03	0.01	3.40	<0.01	0.75	0.34	2.22	<0.05	1.50	0.95	1.58	0.11	0.06	0.16	0.36	0.72

Est. = Estimate; S.E. = Std.Error; Stat. = Statistic; P = p.value.

### 3.3 Mediation analysis of inflammation and nutrition pathways

**Figure 3:**
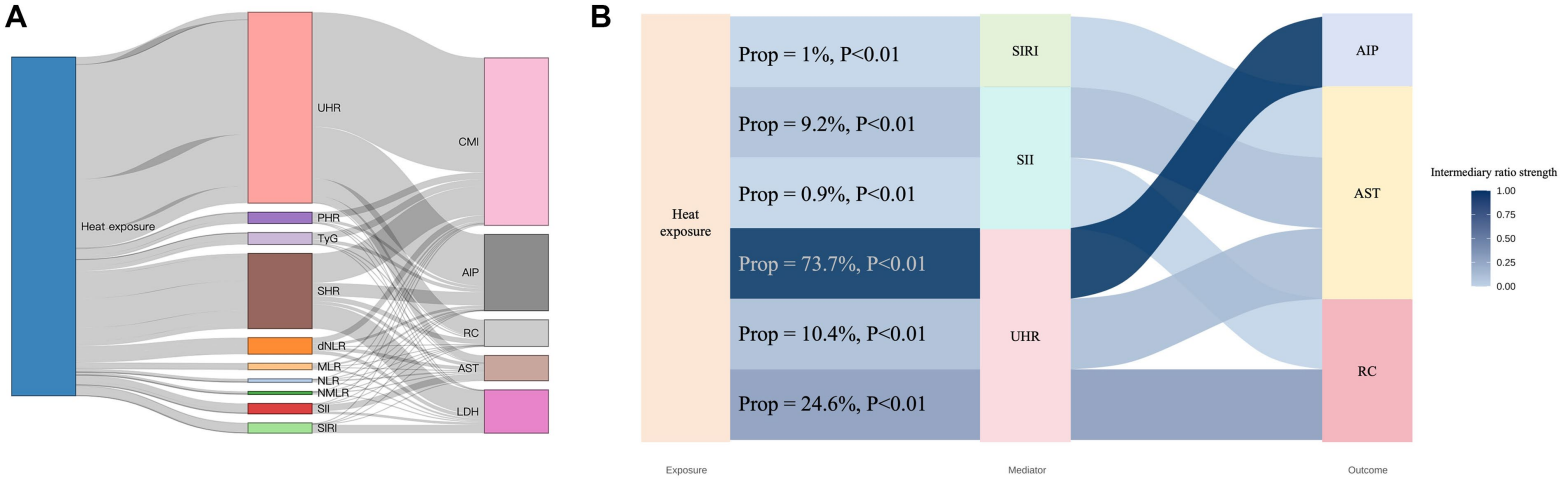
Comparative mediation screening and path analysis of the metabolic–inflammatory axis. **(A)** Proportional contributions of 10 candidate mediators—four metabolic indices (UHR, PHR, TyG, SHR) and six inflammatory indices (MLR, NLR, NMLR, SII, SIRI, dNLR)—to the total effect of CEHWI on various subclinical cardiovascular outcomes (RC, AIP, CMI, AST and LDH). This screening emphasizes the dominant role of the UHR-mediated metabolic–lipid pathway across the injury cascade. **(B)** Mediation path diagram depicting the mediation pathway from cumulative thermal burden to subclinical injury, quantifying the mediating effect of the UHR-mediated metabolic–lipid axis. Values are standardized path coefficients for direct and indirect effects, with mediation proportions. Statistical significance and 95% CIs were estimated by bootstrap (5,000 iterations).

To clarify the pathways linking CEHWI to CV injury, we systematically assessed 10 candidate mediators across metabolic–lipid (UHR, TyG, PHR, SHR) and inflammatory (SII, SIRI, NLR, dNLR, MLR, NMLR) domains (Figure 3A). Although all indices reached statistical significance as mediators (all *p* < 0.01), their contributions to the heat–cardiovascular axis differed substantially. Comparative screening identified UHR as the dominant mediator, significantly exceeding all other candidates (Figure 3B). UHR mediated 73.7% of the total effect of cumulative heat on AIP (*p* < 0.01), 24.6% on RC (*p* < 0.01) and 10.4% on AST (*p* < 0.01). By contrast, inflammatory indices (SII, SIRI) and glucose-related markers (TyG, SHR) had limited mediating effects, typically accounting for less than 12% of the total effect for any outcome. Conventional leukocyte ratios contributed only marginally (0.8–5.5%), indicating that heat-induced cardiovascular strain is conveyed mainly via specific metabolic–lipid remodeling rather than by broad immune activation. Together, these findings point to a UHR-mediated metabolic–lipid axis as the principal route of subclinical injury under cumulative thermal stress.

### 3.4 Vulnerability and sensitivity analyses

**Figure 4:**
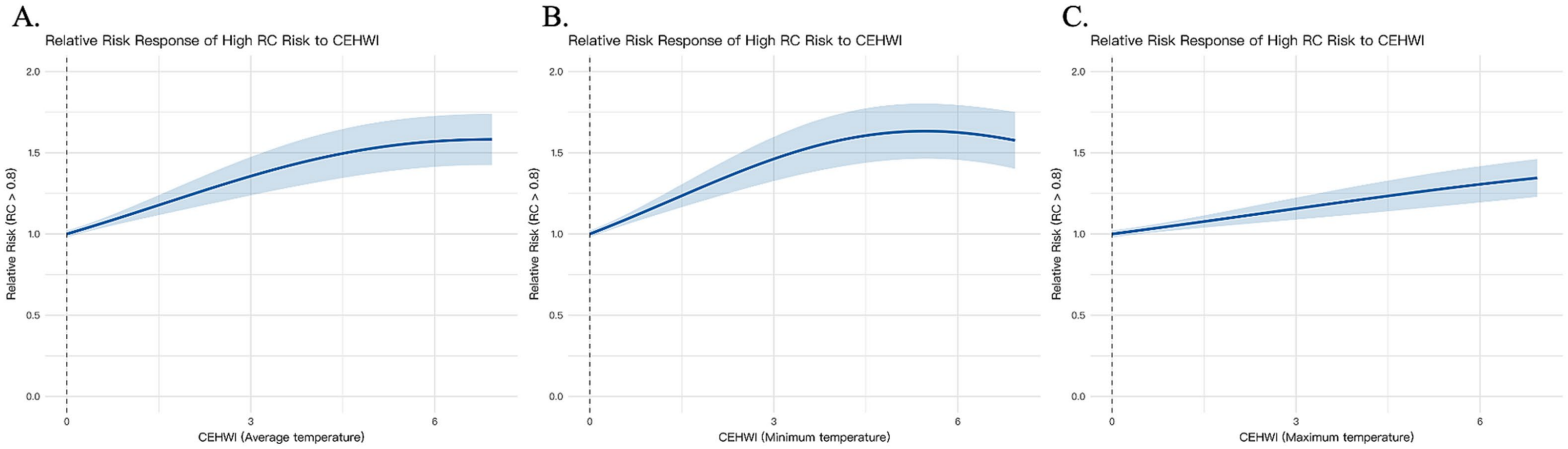
Sensitivity analysis of the association between CEHWI (calculated using mean, minimum, and maximum apparent temperature) and elevated remnant cholesterol (RC > 0.5) risk. The horizontal axis denotes CEHWI level; the vertical axis denotes relative risk (RR) with 95% confidence intervals (shaded).

**Figure 5:**
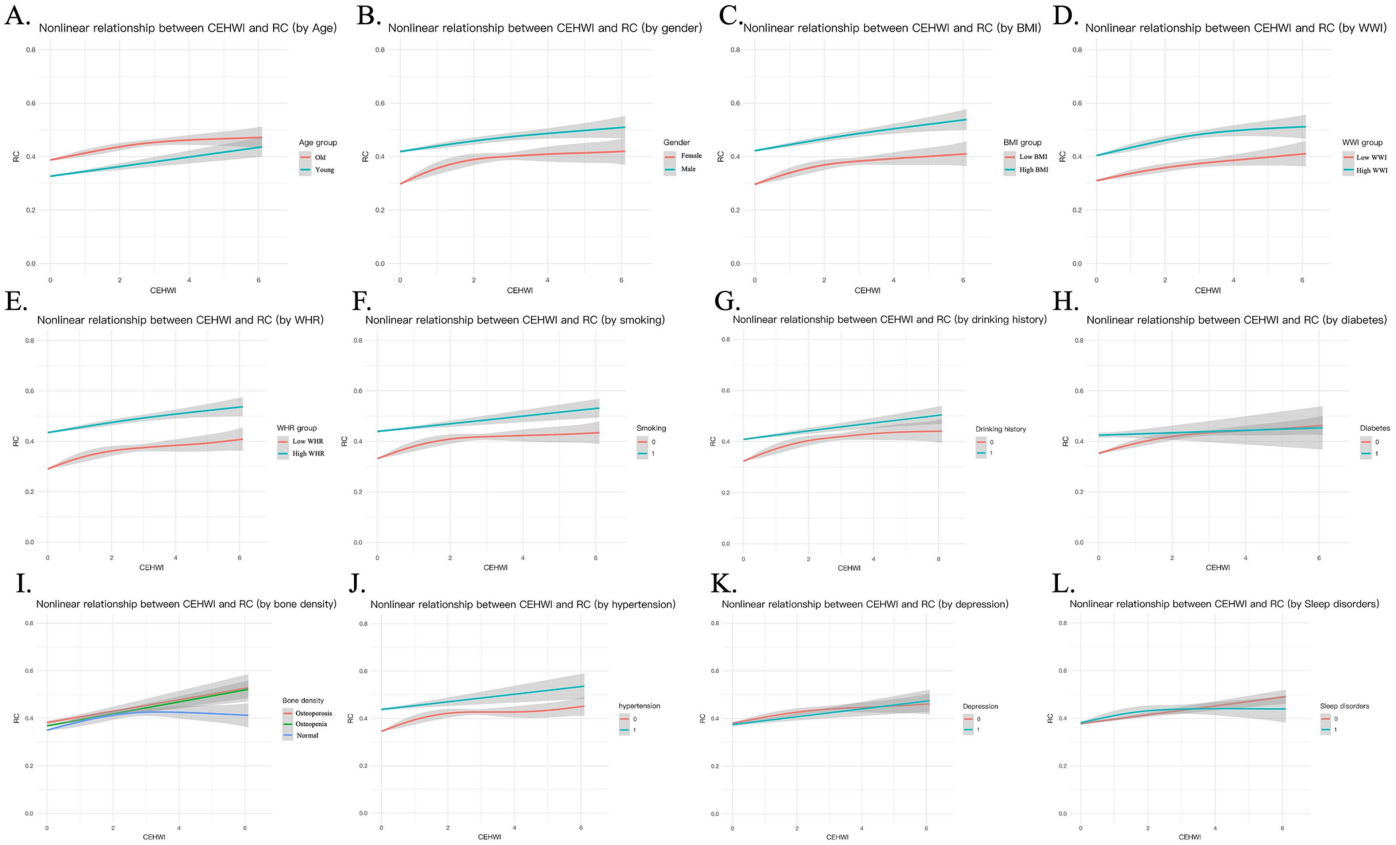
Subgroup analysis of the association between cumulative heat exposure and remnant cholesterol (RC). Coefficient plots **(A–L)** show the adjusted effect estimates (β) and 95% CIs for the association between CEHWI and RC across twelve comparative strata: **(A)** Age, **(B)** Sex, **(C)** BMI, **(D)** WWI, **(E)** WHR, **(F)** Smoking status, **(G)** Alcohol consumption, **(H)** Diabetes, **(I)** Bone mineral density, **(J)** Hypertension, **(K)** Depression, and **(L)** Sleep disorders. Each panel emphasizes the differing RC response to thermal burden between the dichotomized subgroups.

Quantitative analysis revealed a strong positive correlation between CEHWI and RC, with increasing thermal intensity associated with progressively higher RC concentrations (Figure 2). To assess the robustness of our findings, we recalculated CEHWI using alternative temperature metrics. The positive associations persisted in models based on mean, minimum and maximum apparent temperatures (Figure 4), indicating that the cardiovascular strain observed is a stable response to thermal burden regardless of the temperature metric used. In sensitivity analyses across 1-, 3-, 5-, and 7-day windows, associations remained directionally consistent. For atherogenic markers, effect estimates strengthened with longer windows: for AIP, *β* increased from 0.019 (95% CI 0.007–0.031; *p* = 0.002) at 1 day to 0.056 (0.033–0.080; *p* < 0.001) at 7 days; for RC, β increased from 0.088 (0.067–0.109; p < 0.001) to 0.140 (0.099–0.181; p < 0.001). AST showed a positive but less stable pattern across windows (1 day: *p* = 0.133; 3 days: *p* = 0.027; 5 days: *p* = 0.009; 7 days: *p* = 0.093) (Supplementary Figures S1, S2 and Supplementary Table S1). We then performed subgroup analyses to identify susceptible populations (Figure 5). Associations between heat exposure and atherogenic markers (AIP and RC) were significantly amplified in older adults (>60 years) and in individuals with pre-existing hypertension (both *p* < 0.05). Although steeper injury trajectories were also apparent in males, smokers, and in participants with higher anthropometric indices (BMI, WWI or WHR), as well as in those with adverse lifestyle factors (alcohol consumption) or comorbidities such as osteoporosis, the most statistically robust vulnerabilities were concentrated in the older adults and hypertensive groups. This pattern suggests that heat-related cardiovascular risk is chiefly borne by individuals with pre-existing metabolic vulnerability, consistent with the “Double-Hit” hypothesis.

## 4 Discussion

This large-scale retrospective cross-sectional of more than 64,088 participants provides a rigorous epidemiological basis for linking cumulative heat exposure to subclinical CV damage. Using the novel CEHWI, we demonstrate a clear dose–response relationship between thermal burden and early markers of CV injury, including RC and the AIP. Crucially, we identify the UHR as the principal mediator, revealing a previously unrecognized metabolic–inflammatory pathway that connects environmental heat stress to increased cardiovascular vulnerability.

### 4.1 Beyond transient peaks: the biological significance of sustained thermal burden

Traditional environmental cardiology has focused on acute temperature spikes or transient heatwaves as the principal drivers of cardiac risk (8, 9, 17). Our findings, however, support a shift to a “cumulative thermal dose” perspective to capture the broader spectrum of environmental harm. The strong association between CEHWI and higher RC and AIP shows that subclinical cardiovascular injury is not only an acute reaction to thermal shock but a result of prolonged homeostatic strain. Physiologically, sustained heat exposure triggers a series of compensatory responses (from persistent peripheral vasodilation to increased cardiac workload) that exhaust physiological reserves and eventually lead to metabolic failure (8, 42–45). This cumulative effect is reflected in a progressive progression of injury: beginning with the increase of subclinical atherogenic strain (RC, AIP) (35, 36) and then the increase of myocardial injury markers (AST, LDH) following sustained exposure (46, 47). This duration–intensity dependence implies that “invisible damage” from heat begins well before the sudden clinical events (such as myocardial infarction) are visible. We think of heat exposure as a sustained stressor rather than a transient spike, which provides a biological basis for earlier risk stratification in warming climate.

### 4.2 UHR: the metabolic-inflammatory nexus and cellular stress trigger

Identifying UHR as the principal mediator—accounting for 73.7% of AIP variation—is the study’s most important conceptual advance. UHR is not merely a clinical ratio; it embodies the intersection of purine metabolism and reverse cholesterol transport (24, 38). Under heat stress, systemic dehydration and renal hypoperfusion raise serum uric acid (48), a strong intracellular pro-oxidant (49), and thermal stress simultaneously lowers HDL-C through lipid remodeling and hormonal stress (50–52). Importantly, this UHR driven axis links population-level observations to molecular sensors of environmental stress (53). Elevated uric acid and lipid dysregulation are known to disrupt proteostasis and may trigger unfolded protein response (UPR) in the vascular endothelium (54–57). We infer that that heat-induced increases in UHR creates a maladaptive thermal–metabolic environment that undermines homeostatic resilience of the coronary microcirculation. In the vulnerable ischemic recovery, this coupling of metabolic and environmental stressors likely activates intracellular stress sensing networks. Our findings suggest that UHR-driven metabolic stress activates conserved cell stress sensing networks and disrupts proteostasis in the coronary microcirculation, offering a mechanistic hypothesis for further experimental validation. This hypothesis provides a clear clinical rationale for investigating the molecular pathways that mediate heat-related cardiovascular damage.

### 4.3 Vulnerability and the “double-hit” hypothesis: a nuanced perspective

Our subgroup analyses indicated that older adults and those with pre-existing metabolic syndrome or hypertension are more susceptible to heat. This phenomenon can be conceptually framed through a “Double-Hit” model: baseline metabolic fragility constitutes the first hit by reducing physiological reserves, and cumulative heat exposure forms the second hit, triggering a maladaptive cascade of elevated UHR and lipid dysregulation. For males and in smokers, the higher risk likely reflects higher baseline oxidative stress and impaired endothelial function (58–61), which exacerbate the UHR-mediated injury pathway. Notably, hypertension markedly increases the risk of heat-induced metabolic collapse, as shown by the more rapid rise in RC and AIP among hypertensive subjects compared with normotensive controls. This sensitivity implies that hypertension functions as a priming factor, lowering the threshold at which environmental stressors disrupt lipid homeostasis. Although long-term antihypertensive therapy (for example, ACE inhibitors or beta-blockers) may confer some structural protection (8), our results indicate that hypertensive individuals remain metabolically hyper-responsive to thermal strain. Consequently, clinical management must extend beyond blood pressure control. Precision prevention — including targeted metabolic monitoring and sustained cooling interventions — is therefore essential to reduce cumulative heat-related risk in these vulnerable populations.

Our subgroup analyses indicated that older adults and people with metabolic syndrome or hypertension are more vulnerable to heat exposure. This can be conceptualized as a Double Hit: baseline metabolic fragility is the first hit by reducing physiological reserves, cumulative heat exposure is the second hit by creating high UHR and lipid dysregulation. For males and smokers, the higher risk is likely due to higher baseline oxidative stress and impaired endothelial function (58–61), which further aggravate UHR injury pathway. Hypertension increases the risk of heat-induced metabolic collapse significantly as shown by faster rise in RC and AIP among hypertensive subjects than normotensive controls. This implies that hypertension functions as a priming mechanism lowering the threshold at which environmental stressors disrupt the lipid homeostasis. Longterm antihypertensive therapy (e.g., ACE inhibitors or betablockers) may provide some structural protection (8), however our results suggest that hypertensive individuals remain metabolically hyperresponsive to thermal strain. Thus clinical management must be extended beyond blood pressure control. Precision prevention — targeted metabolic monitoring and sustained cooling intervention — must also be used to reduce cumulative heat related risk in these vulnerable populations.

### 4.4 Strengths and limitations

A key strength of this study is the sample size and the novel use of CEHWI, which captures the multidimensional and cumulative nature of thermal stress and thus transcends single temperature peaks limitations. These window-based sensitivity analyses support the robustness of our primary 7-day specification while suggesting that temporal accumulation effects are stronger and more consistent for lipid-related endpoints (AIP, RC) than for AST. However, several limitations must be acknowledged. First, although the cross-sectional design precludes definitive causal inference, the robust dose–response and duration-dependent effects provide quasi-temporal evidence supporting a heat-induced injury model. Moreover, the biomarkers used (RC, AIP, LDH, AST) do not directly quantify structural damage (e.g., myocardial necrosis or plaque rupture); future imaging or troponin studies are needed to validate injury interpretation. Second, the absence of detailed information on individual adaptive behaviors (for example, air-conditioning use or time spent outdoors) may cause exposure misclassification, which would typically bias estimates towards the null. Third, although our multivariable models employed high-resolution data to adjust rigorously for a comprehensive suite of ambient air pollutants (PM_2.5_, PM_10_, O_3_, NO_2_ and SO_2_), we did not explicitly model potential synergistic interactions between thermal strain and chemical co-exposures. Future studies using environmental-mixture frameworks are therefore warranted to disentangle the cumulative ‘double burden’ of heat and pollution on metabolic remodeling. Fourth, although we adjusted for a wide range of covariates, residual confounding by unmeasured factors—such as precise dietary patterns—cannot be ruled out entirely. Finally, because our cohort is concentrated in central and western China, the geographic and socioeconomic variability of heat-related effects requires confirmation in multi-center studies spanning diverse climatic regions.

### 4.5 Clinical translation and environmental health policy

These findings have substantial clinical and public-health implications as extreme climatic events become more frequent and prolonged. First, we propose UHR as a “biological thermometer” of cardiovascular risk: integrating it into routine health screening would provide an early metabolic window to identify individuals at risk before overt clinical events occur. Second, our results indicate that cooling interventions should be maintained for the duration of a heatwave, not just during peak hours, to reduce cumulative physiological strain. In communities with a high burden of metabolic diseases, protecting vulnerable groups (e.g., older adults, hypertension) with access to cooling infrastructure should be priority. Adding metabolic biomarkers such as UHR to heat–health early warning systems could change climate adaptation strategies by preventing subclinical injury and reducing the growing cardiovascular burden in an increasingly warm world.

### 4.6 Future directions

Translating these epidemiological insights into clinical impact requires targeted molecular validation. Future work should focus on whether UHR-mediated “metabolic hit” activates conserved homeostatic stress signals (for example intracellular sensors controlling proteostasis) in the cardiac microvasculature. How thermal strain affects these networks is important to understand how heat exposure causes myocardial injury and post-ischemic repair. Mapping these stress pathways is the next step from observation to precise therapeutic action and targets to preserve heart resilience in warming world.

## 5 Conclusion

This study demonstrates that short-term cumulative heat exposure is associated with subclinical cardiovascular injury, particularly in individuals with pre-existing metabolic fragility. UHR merged as the predominant mediator in this associational pathway, defining a previously unknown metabolic–inflammatory link between environmental stress and subclinical vascular damage. These results argue for a shift in environmental cardiology away from reactive monitoring of acute heat spikes towards proactive, individual-level assessment of cumulative thermal burden. In this context UHR is a “biological thermometer” providing a measure of physiological strain early and integrated measure of physiological stress. In this sense, metabolic biomarkers like UHR can be used as early warning systems and tailor interventions to high risk individuals are public health priorities to reduce the increasing cardiovascular burden in a warming world.

## Data Availability

The raw data supporting the conclusions of this article will be made available by the authors, without undue reservation.

## Supplementary material

SUPPLEMENTARY FIGURE S1 Forest plot of CEHWI-outcome associations across 1-, 3-, 5-, and 7-day exposure windows.

SUPPLEMENTARY FIGURE S2 Heatmap of effect estimates (β) for CEHWI-outcome associations across different exposure windows.

## Glossary

CV: Cardiovascular
CEHWI: Cumulative Excess Heat Wave Index
AT: Apparent Temperature
RC: Remnant Cholesterol
AIP: Atherogenic Index of Plasma
AST: Aspartate Aminotransferase
LDH: Lactate Dehydrogenase
UHR: Uric Acid-to-HDL Cholesterol Ratio
SIRI: Systemic Inflammation Response Index
SII: Systemic Immune–Inflammation Index
TyG: Triglyceride-Glucose Index
PHR: Prealbumin-to-HDL cholesterol Ratio
BMD: Bone Mineral Density
DXA: X-ray Absorptiometry
BMI: Body Mass Index
CIs: Confidence Intervals
WWI: Waist-to-Weight Index
WHR: Waist-to-Hip Ratio
CMI: Cardio-metabolic Index
UPR: Unfolded Protein Response
RR: Relative risk
