# Supplementary figures and images for: UHR as a systemic sensor of cumulative heat exposure and subclinical cardiovascular injury: evidence from 64,088 adults
Source: Front Public Health. 2026 May 7;14:1825331. doi: 10.3389/fpubh.2026.1825331 (PMC13190384; doi:10.3389/fpubh.2026.1825331)

Forest Plot by Outcome and Exposure Window

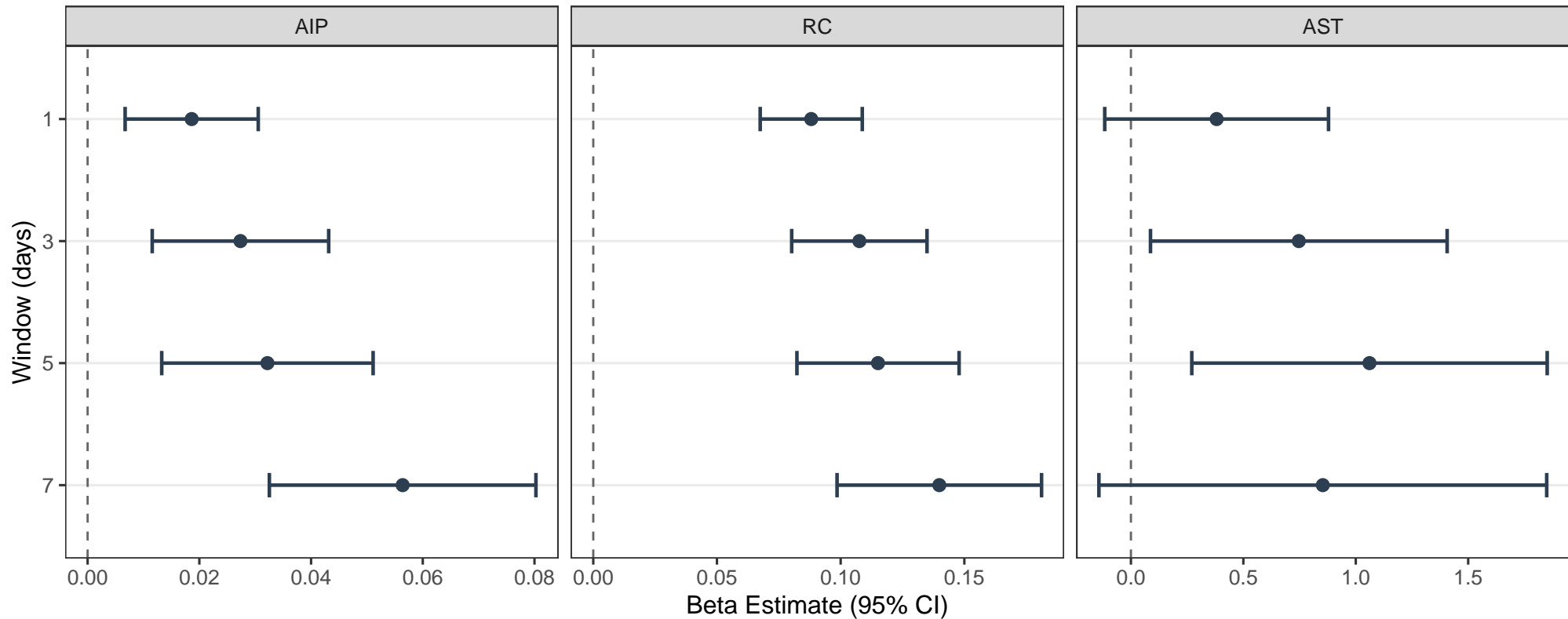

Supplement: SUPPLEMENTARY FIGURE S1 — Forest plot of CEHWI-outcome associations across 1-, 3-, 5-, and 7-day exposure windows. [file Data_Sheet_1.PDF]

# Sensitivity Heatmap of Effect Estimates

Beta

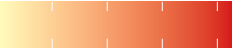

0.25 0.50 0.75 1.00

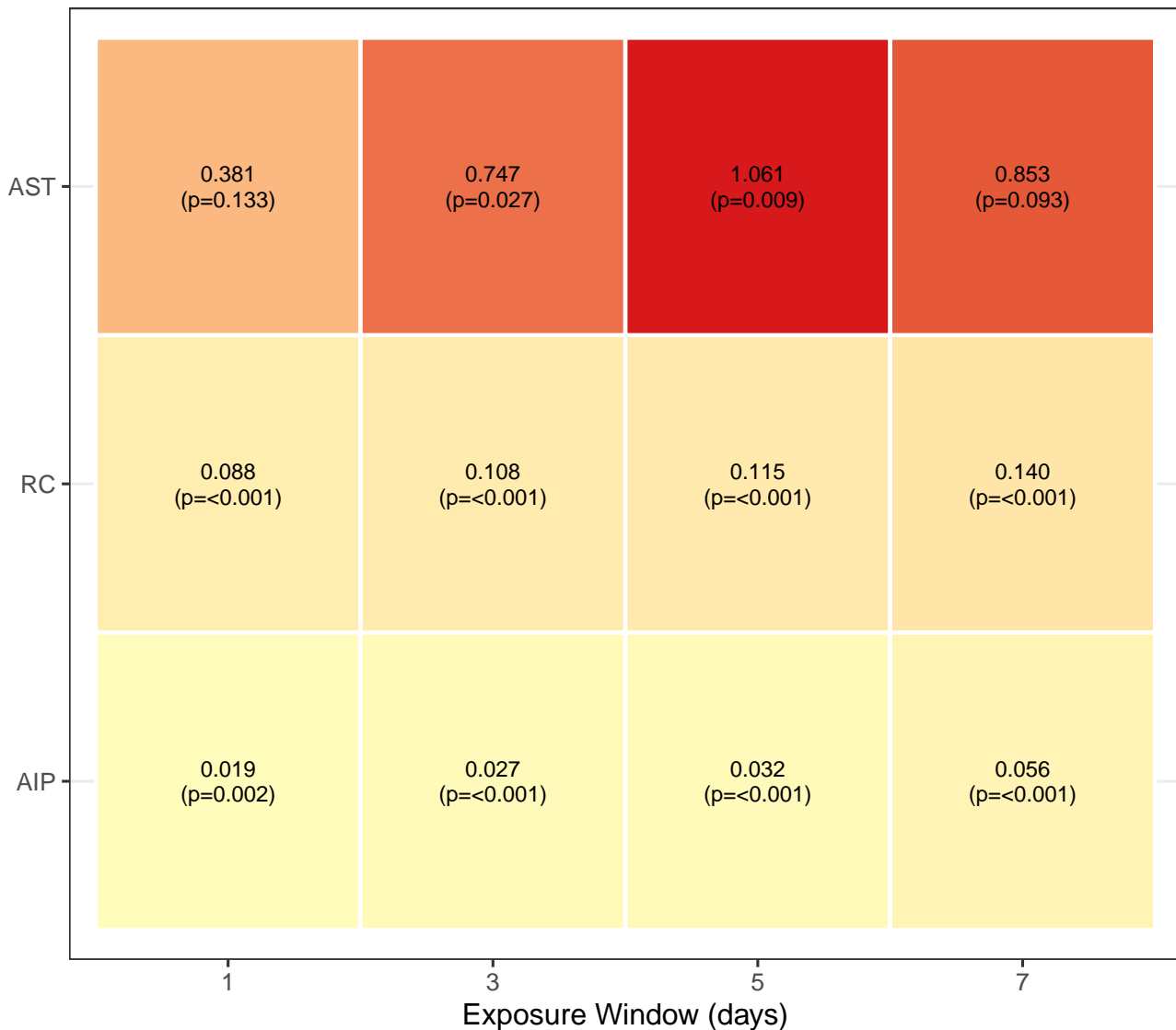

Supplement: SUPPLEMENTARY FIGURE S2 — Heatmap of effect estimates (β) for CEHWI-outcome associations across different exposure windows. [file Data_Sheet_2.PDF]
